# Pyridine-4-thiol as halo­gen-bond (HaB) acceptor: influence of the noncovalent inter­action in its reactivity

**DOI:** 10.1107/S205322962300205X

**Published:** 2023-03-15

**Authors:** Marta E. G. Mosquera, Silvia Dortez, Francisco Fernández-Palacio, Pilar Gómez-Sal

**Affiliations:** a Department of Organic and Inorganic Chemistry, Institute of Chemical Research ‘Andrés M. del Río’ (IQAR), Universidad de Alcalá, Campus Universitario, 28871 Alcalá de Henares, Madrid, Spain; b Department of Analytical Chemistry, Physical Chemistry and Chemical Engineering, Institute of Chemical Research ‘Andrés M. del Río’ (IQAR), Universidad de Alcalá, Campus Universitario, 28871 Alcalá de Henares, Madrid, Spain; University of Sheffield, United Kingdom

**Keywords:** halo­gen bond, HaB, pyri­dine-4-thiol, C—Cl bond activation, crystal structure, chalcogen bond

## Abstract

The study of pyri­dine-4-thiol as a halo­gen-bond (HaB) acceptor has allowed the isolation of its cocrystal with the HaB donor IC_6_F_4_I, namely, 1,2,4,5-tetra­fluoro-3,6-di­iodo­benzene bis­(pyri­din-1-ium-4-ylsul­fanide), C_6_F_4_I_2_·2C_5_H_5_NS (**1**), where the S atom is the HaB acceptor, while the pyri­dine position is blocked by the proton. Furthermore, the S atom acts a dual acceptor and also establishes an inter­action with the pyri­dinium proton from an adjacent mol­ecule. The presence of these inter­actions in **1** contributes to the stabilization of the zwitterionic form. This pre-organization seems to have an influence on the reactivity of the com­pound since when left in di­chloro­methane solution, an unusual activation of the C—Cl bond is observed that leads to the formation of the bis­[(pyri­din-1-ium-4-yl)sul­fan­yl]methane dication, while the Cl atoms are still present as chloride counter-ions, *i.e.* 4,4′-[methane­diyldi(sul­fanedi­yl)]dipyri­dinium dichloride, C_11_H_12_N_2_S_2_^2+^·2Cl^−^ (**2**). In the crystal structure of **2** it is observed that the S atom is now acting as the donor part of a chalcogenide bond with the chloride anions.

## Abstract 2

Pyridine-4-thiol has been explored as an acceptor for a halogen-bond (HaB) inter­action. For this com­pound, it is the S atom instead of the pyri­dine group that is the moiety of choice for establishing the inter­actions, producing a betaine-type structure. Inter­estingly, when left in solution, this com­pound forms a methyl­ene-bridged sulfide that implies the activation of the CH_2_Cl_2_ solvent. The pre-organization imposed by the HaB seems to have a clear influence on the outcome.

## Introduction

Halogen bonding (HaB) is a noncovalent inter­action that has attracted increasing attention during the last few decades, due to its role in crystal engineering, mol­ecular recognition pro­cesses, as a structure-directing tool and for the modulation of some physical properties (Politzer *et al.*, 2007 ▸; Brammer *et al.*, 2001 ▸; Aakeröy *et al.*, 2013 ▸; Cavallo *et al.*, 2016 ▸). Also, its influence in reactivity and catalysis is increasingly acknowledged (Bulfield & Huber, 2016 ▸; Bamberger *et al.*, 2019 ▸; Jónsson *et al.*, 2022 ▸).

In our group, we have explored this inter­action in metallic com­plexes, both for main group and transition metals, and have observed the influence of HaB in the structural arrangement and reactivity (Dortéz *et al.*, 2020 ▸; Mosquera *et al.*, 2016 ▸, 2017 ▸; Vidal *et al.*, 2013 ▸). In this work, we are inter­ested in exploring pyri­dine-4-thiol as a HaB acceptor since there are two possible sites able to act as such, namely, the pyri­dine group and the S atom. Pyridines are one of the most popular types of HaB acceptor, a search in the Cambridge Structural Database (CSD; Version 5.42, with updates to September 2021; Groom *et al.*, 2016 ▸) for HaB inter­actions with C—*X* (*X* = Br or I) gives over 1000 hits. On the other hand, sulfur has two available lone pairs and can be a potent HaB acceptor since it can inter­act with more than one HaB donor, which can lead to inter­esting topologies. However, it has been less studied, although in a search in the CSD, there is a significant number of structures that display S atoms as a HaB acceptor; a search for C—*X*⋯S (*X* = Br or I) inter­actions in the angle range 160–180° gave 715 hits.

We have explored the inter­action of pyri­dine-4-thiol with the iconic IC_6_F_4_I mol­ecule as HaB donor. Inter­estingly, for this mol­ecule, it is not the pyri­dine group but the S atom that acts as the HaB acceptor, which is surprising since in the related com­pound Py–S–Py, it is the pyri­dine group that acts as the HaB acceptor (Arman *et al.*, 2010 ▸). Furthermore, the presence of C—S⋯I inter­actions in this cocrystal favours the stabilization of the zwitterionic form for pyri­dine-4-thiol (namely, pyri­din-1-ium-4-ylsul­fanide), which has important implications in the reactivity of the mol­ecule, and in fact an unusual C—Cl activation of the di­chloro­methane solvent is observed to give the bis­[(pyri­din-1-ium-4-yl)sul­fan­yl]methane dication.

The structures of 1,2,4,5-tetra­fluoro-3,6-di­iodo­benzene bis­(pyri­din-1-ium-4-ylsul­fanide), **1**, and 4,4′-[methane­diyldi(sul­fanedi­yl)]dipyri­dinium dichloride, **2**, are reported.

## Experimental

### General considerations

All manipulations were carried out under an inert atmos­phere of argon using standard Schlenk techniques. All sol­vents were dried prior to use following standard methods. All reagents were commercially obtained and were used without further purification.

### Synthesis and crystallization

#### Synthesis of cocrystal 1

Pyridine-4-thiol (14 mg, 0.126 mmol) and 1,2,4,5-tetra­fluoro-3,6-di­iodo­benzene (50 mg, 0.126 mmol) were placed in a Schlenk flask. To this mixture, CH_2_Cl_2_ (25 ml) and tetra­hydro­furan (12 ml) were added. In order to com­pletely dissolve the solid, a brief reflux with a heat gun was performed. The solution was filtered and left to evaporate slowly. After three weeks, yellow crystals were isolated (yield: 63%, 40 mg).

#### Synthesis of (HPy–CH_2_–PyH)Cl_2_ (2)

To pyri­dine-4-thiol (50 mg, 0.45 mmol) in a Schlenk flask was added CH_2_Cl_2_ (10 ml). To this suspension, C_6_F_4_I_2_ (180 mg, 0.45 mmol) was added and, to dissolve the mixture com­pletely, tetra­hydro­furan (10 ml) were added. The solution was left to stir for 48 h. A clear solution was formed, to which a layer of hexane (16 ml) was added. After the solution had dif­fused into the hexane, brown crystals of **2** of good enough quality for analysis by X-ray dif­fraction were isolated (yield: 32%, 22 mg).

### Refinement

Crystal data, data collection and structure refinement details are summarized in Table 1 ▸. H atoms were placed geometrically and left riding on their parent atoms, except for the H atoms bonded to N1 in cocrystal **1**, and C10 and N1 in salt **2**, which were found in a Fourier map.

## Results and discussion

**Figure 2:**
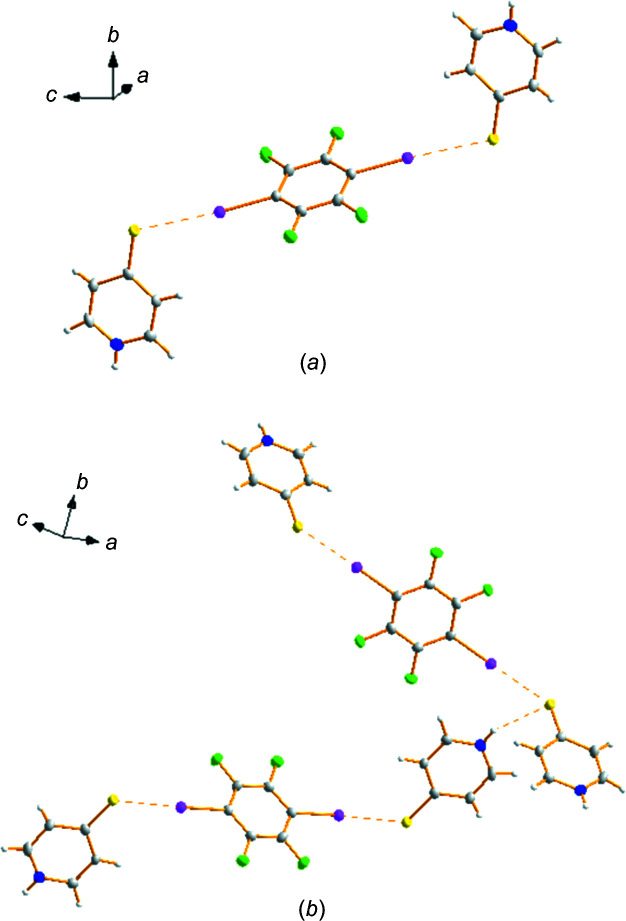
Displacement ellipsoid plot (30% probability) of **1**, showing (*a*) the C—I⋯S halo­gen bonding and (*b*) a view of the mol­ecular packing *via* halo­gen and hydro­gen bonding (dashed lines).

In order to achieve the crystallization of pyri­dine-4-thiol and IC_6_F_4_I, both com­pounds were dissolved in a mixture of CH_2_Cl_2_ and tetra­hydro­furan under argon. The suspension was heated briefly to obtain a clear solution (Fig. 2 ▸). From this, orange crystals were isolated after three weeks by slow evaporation and the structure was determined by X-ray dif­fraction analysis.

As shown in Fig. 2 ▸, a cocrystal has been formed incorporating two mol­ecules of pyri­dine-4-thiol and one of IC_6_F_4_I. This com­pound crystallized in the monoclinic space group *P*2_1_/*c*. In this case, it is the S atom that acts as the HaB acceptor, which stabilizes the zwitterionic form of pyri­dine-4-thiol and, at the same time, the pyri­dine group is blocked by the proton. It should be noted that pyri­dine-4-thiol has been used frequently as a ligand for *d*- and *f*-block metals, and in those cases most often the zwitterionic form is the one present (CSD; Groom *et al.*, 2016 ▸).

**Figure 3:**
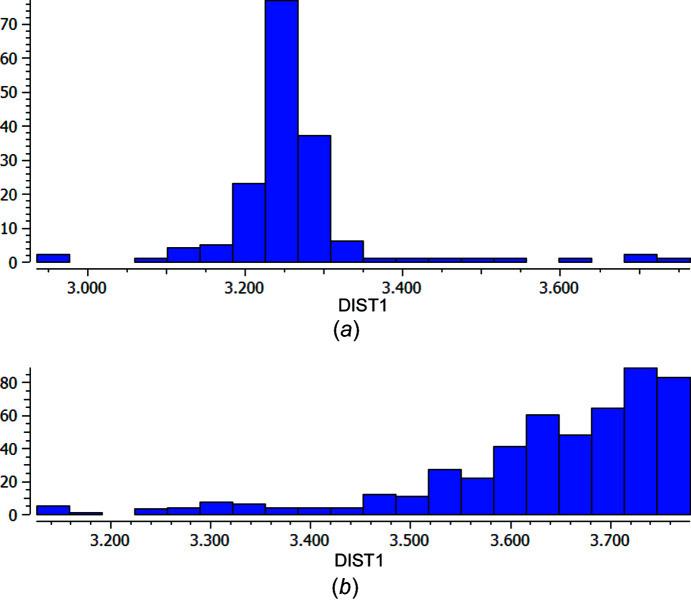
Histograms of the S⋯I distances for the S⋯I—C inter­action for (*a*) an S atom with a −1 charge (149 hits) and (*b*) an S atom with no charge (339 hits). In salt **2**, the S⋯I distance is 3.158 (7) Å.

For the HaB established between the pyri­dine-4-thiol and the perfluorinated unit, the S⋯I distance is particularly short [3.158 (7) Å and C—I⋯S = 175.74 (5)°; Table 2 ▸]; considering that the van der Waals radius sum is 3.78 Å (Bondi, 1964 ▸), this inter­action implies a significant reduction of 16.4%. This short distance has also been observed in other com­pounds and, in all cases, the S atom has a negative charge, as in ours, as evidenced by a search in the CSD. As shown in Fig. 3 ▸(*b*), when the S atom is neutral, the majority of the inter­actions appear at S⋯I distances over 3.5 Å. For shorter distances [Fig. 3 ▸(*a*)], the majority of the examples imply the thyo­cyanate anion, SCN^−^ (Zuluaga *et al.*, 2020 ▸; Postnikov *et al.*, 2015 ▸; Bozopoulos & Rentzeperis, 1986 ▸; Hirschberg *et al.*, 2014 ▸).

Sulfur can form bifurcated inter­actions and in this case behaves as such; on one side it inter­acts with the I atom and on the other side with the pyri­dinium N—H proton. This behaviour as a dual acceptor for both a halo­gen and a H atom has been observed previously; however, there are not many examples (Ding *et al.*, 2020 ▸).

The perfluorinated unit, *p*-C_6_F_4_I_2_, inter­acts from both sides to give discrete units formed by the inter­action of two mol­ecules of pyri­dine-4-thiol. These units are linked *via* weaker N—H⋯I and C—H⋯F inter­actions to give layers. As mentioned before, for the related Py–S–Py derivative, the inter­action is established between the pyri­dine group and the I atom to give an N_Py_⋯I—C contact (N_Py_⋯I = 2.838 Å and N_Py_⋯I—C = 177.93°) (Arman *et al.*, 2010 ▸). Hence, the preference for one group or the other is not very strong and may even be governed by kinetics and, if there is a com­peting aceptor, such is in the case of the proton, may direct the choice of the donor. In fact, for the thio­cyanate, which can also act as a dual donor, the formation of both HaB inter­actions with the N or the S atom has also been oberved for the same donors (Soldatova *et al.*, 2020 ▸).

When the reaction is performed on a larger scale and left stirring for 48 h, the solution changes colour to brown. It was possible to obtain crystals from this solution by dif­fusion into a hexane layer. The isolated brown crystals were of good enough quality to be determined by single-crystal X-ray dif­fraction. Inter­estingly, a methyl­ene-bridged sulfide resulting from the displacement of both Cl atoms from di­chloro­methane by two mol­ecules of pyri­dine-4-thiol, *i.e.* an [*L*_2_CH_2_]^2+^ dication, is formed. The displaced Cl atoms are still present as chloride counter-ions of the dication generated. The activation of the C—Cl bond has been observed previously, most often *via* a radical mechanism and less frequently *via* a nucleophilic mechanism. In fact, in our group, we have observed a similar reactivity when a zwitterionic betaine derivative com­posed of a nucleophilic carbene and a carbodimide (CDI-NHC) is left stirring for a long time in a di­chloro­methane solution (Sánchez-Roa *et al.*, 2018 ▸). As in this case, the activation of the C—Cl bond is promoted by the presence of zwitterionic species. As mentioned before, the zwitterionic form of pyri­dine-4-thiol is stabilized by the presence of the HaB inter­action, which implies a pre-organization that influences the outcome of the reaction. A similar behaviour has been described previously for isocyanates (Soldatova *et al.*, 2020 ▸).

Moreover, the evolution of pyri­dine-4-thiol in solution when it is left stirring in solution at 67 °C for 15 h gives the [HS–Py–Py]^+^ derivative, as has been reported previously (Ding *et al.*, 2020 ▸). In our case, the presence of IC_6_F_4_I favours the com­petitive reaction that implies the CH_2_Cl_2_ activation.

**Figure 4:**
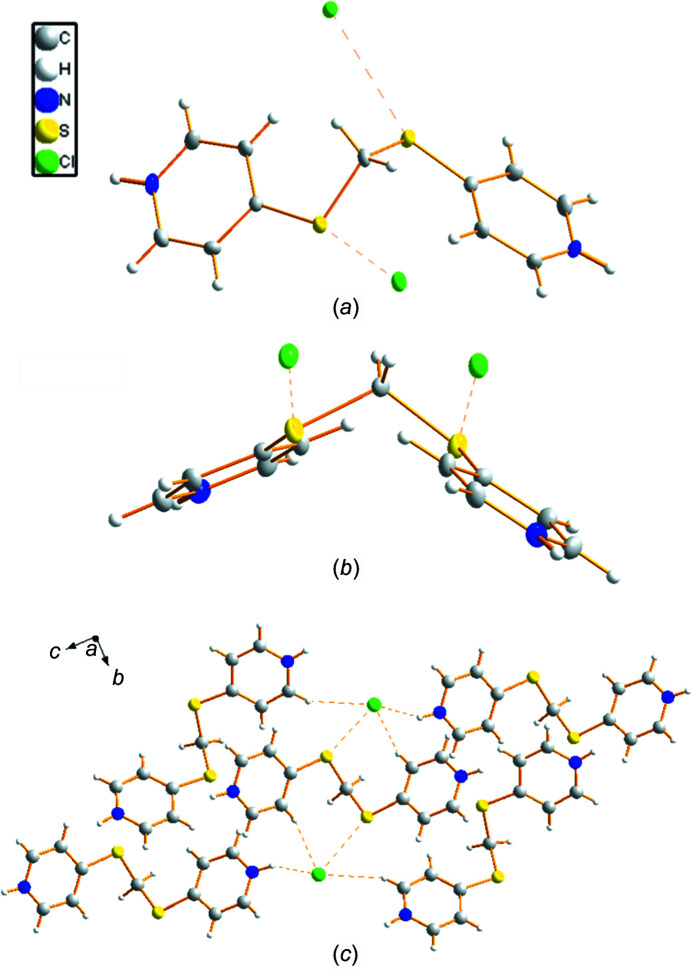
Displacement ellipsoid plot (30% probability) of **2**, showing (*a*) a view along the *bc* plane, (*b*) a view along the *a* direction and (*c*) a view of the packing directed by hydro­gen and chalcogen bonds.

Compound **2** crystallized in an ortho­rhom­bic noncentrosymmetric spatial group *Fdd*2. The central C atom shows an angle slightly wider [116.3 (4)°] than that typical of *sp* ^3^-hy­bridation, probably due to the presence of the S atoms; the value is similar to that previously reported for bis­[(pyri­din-4-yl)sul­fan­yl]methane (Carballo *et al.*, 2007 ▸, 2008*a* ▸,*b* ▸, 2009 ▸; Lago *et al.*, 2013 ▸, 2014 ▸). The S—CH_2_—S plane forms an angle of 78.66 (8)° with the planes of the pyri­dine rings, which are placed in opposite directions relative to the central CH_2_ unit; this arrangement is also observed in the reported derivatives where bis­[(pyri­din-4-yl)sul­fan­yl]methane behaves as a ligand (Carballo *et al.*, 2007 ▸, 2008*a* ▸,*b* ▸, 2009 ▸; Lago *et al.*, 2013 ▸, 2014 ▸). This arrangement is the origin of the conformational chirality shown by this com­pound (Fig. 4 ▸). In our case, this disposition is also influenced by the presence of a hydro­gen bond involving one chloride anion and the proton bonded to atom C4 of the ring. This conformation is reinforced by a chalcogen bond established between the Cl and S atoms [S⋯Cl = 3.373 (4) Å and C—S⋯Cl = 156.94 (5)°], which is significantly shorter than the van der Waals radii radius sum (3.55 Å; Bondi, 1964 ▸). Although the S atom can present two σ-holes, in this case, only one inter­action is observed with the chloride anion.

**Figure 5:**
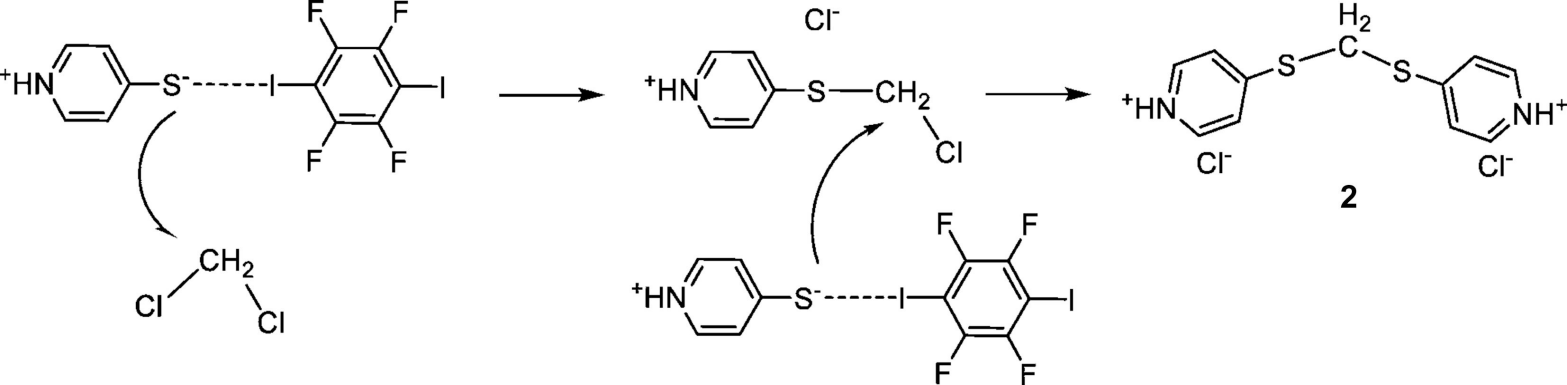
The proposed mechanism for the formation of salt **2**.

As mentioned before, the formation of the cation in **2** is an unusual reaction. Although similar substitution reactions have been reported for some secondary aliphatic (Souquet *et al.*, 1993 ▸), alicyclic (Matsumoto *et al.*, 1984 ▸) or heteroaromatic amines (Juliá *et al.*, 1982 ▸; Zhao *et al.*, 2011 ▸), the process usually takes place under special conditions, such as very high pressures or in highly polar solvent mixtures. Futhermore, it should be noted that the formation of the neutral equivalent bis­[(pyri­din-4-yl)sul­fan­yl]methane from pyri­dine-4-thiol in the presence of CH_2_Cl_2_ has been described previously in basic conditions, *i.e.* in an alcoholic solution with an excess of NaOH, the reaction taking several days to com­plete (Amoedo-Portela *et al.*, 2005 ▸). In our case, the mechanism is dif­ferent, as shown in the reaction pathway displayed in Fig. 5 ▸. Taking into account our previous studies, we have detected that the first step that implies the substitution of the chloride is the rate-determining step, and it proceeds *via* an S_N^2^_ mechanism. This process leads to a reactive chloro­methyl inter­mediate –CH_2_Cl^+^ which evolves quickly to the final product where both Cl atoms have been displaced (Sánchez-Roa *et al.*, 2018 ▸). Hence, in this case, the IC_6_F_4_I role would be as a catalyst.

## Conclusions

The substituted pyri­dine pyri­dine-4-thiol can behave as a HaB acceptor, having a preference for the S atom as the acceptor. In the cocrystal obtained, the concomitant presence of a HaB and a hydro­gen bond contributes to the stabilization of the zwitterionic form of pyri­dine-4-thiol. This arrangement has an effect on the reactivity and the activation of CH_2_Cl_2_ is pro­moted to give the bis[­(4-pyri­din-1-ium-4-yl)sulfanyl]­methane dication, where two sulfide–pyri­dinium units are bonded to a –CH_2_ group. This moiety could only come from the activation of the di­chloro­methane present as a solvent. Inter­estingly, there are not many examples of this type of activity and the current study opens up the possibility of developing processes for the transformation of chorinated hydrocarbons.

## Supplementary Material

Crystal structure: contains datablock(s) global, 1, 2. DOI: 10.1107/S205322962300205X/qz3003sup1.cif

Structure factors: contains datablock(s) 1. DOI: 10.1107/S205322962300205X/qz30031sup2.hkl

Structure factors: contains datablock(s) 2. DOI: 10.1107/S205322962300205X/qz30032sup3.hkl

Supporting information file. DOI: 10.1107/S205322962300205X/qz30031sup4.cml

Supporting information file. DOI: 10.1107/S205322962300205X/qz30032sup5.cml

CCDC references: 2222766, 2222767

## Figures and Tables

**Figure 1:**
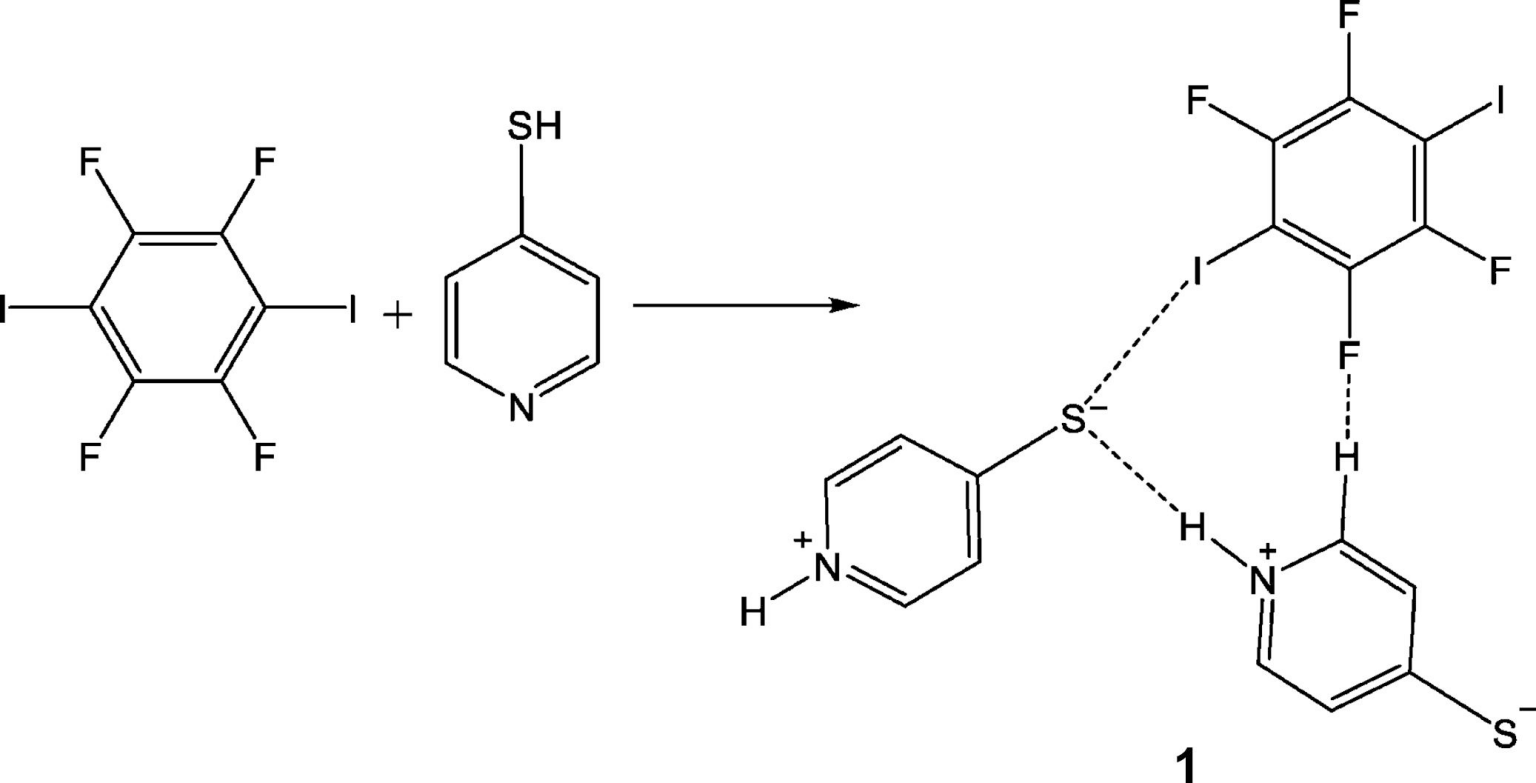
The synthetic scheme for the preparation of cocrystal **1**.

**Table 1:** Experimental details Experiments were carried out at 200 K with Mo *K*α radiation using a Nonius KappaCCD dif­fractometer. H atoms were treated by a mixture of independent and constrained refinement.

	**1**	**2**
Crystal data		
Chemical formula	C_6_F_4_I_2_·2C_5_H_5_NS	C_11_H_12_N_2_S_2_^2+^·2Cl^−^
*M*_r_	624.18	307.25
Crystal system, space group	Monoclinic, *P*2_1_/*c*	Orthorhombic, *F*2*d* *d*
*a*, *b*, *c* (Å)	16.288 (8), 5.790 (4), 10.546 (9)	8.0815 (4), 17.2161 (5), 18.9254 (12)
α, β, γ (°)	90, 104.77 (3), 90	90, 90, 90
*V* (Å^3^)	961.7 (12)	2633.1 (2)
*Z*	2	8
μ (mm^−1^)	3.53	0.79
Crystal size (mm)	0.21 × 0.18 × 0.15	0.4 × 0.3 × 0.25

Data collection		
Absorption correction	Multi-scan (Blessing, 1995 ▸)	–
*T*_min_, *T*_max_	0.371, 0.426	–
No. of measured, independent and observed [*I* > 2σ(*I*)] reflections	3983, 2181, 1458	4579, 1386, 1177
*R*_int_	0.040	0.069
(sin θ/λ)_max_ (Å^−1^)	0.649	0.650

Refinement		
*R*[*F* ^2^ > 2σ(*F* ^2^)], *wR*(*F* ^2^), *S*	0.032, 0.069, 0.93	0.041, 0.101, 1.10
No. of reflections	2181	1386
No. of parameters	122	84
No. of restraints	0	1
Δρ_max_, Δρ_min_ (e Å^−3^)	0.99, −1.09	0.35, −0.30
Absolute structure	–	Flack *x* determined using 445 quotients [(*I* ^+^) − (*I* ^−^)]/[(*I* ^+^) + (*I* ^−^)] (Parsons *et al.*, 2013 ▸)
Absolute structure parameter	–	−0.01 (11)

Computer programs: *COLLECT* (Nonius, 2004 ▸), *DIRAX*/*LSQ* (Duisenberg *et al.*, 2003 ▸), *EVALCCD* (Duisenberg *et al.*, 2003 ▸), *SHELXT2018* (Sheldrick, 2015*a* ▸), *SHELXS2013* (Sheldrick, 2008 ▸), *SHELXL2014* and *SHELXL2016* (Sheldrick, 2015*b* ▸), *ORTEP-3 for Windows* (Farrugia, 2012 ▸) and *WinGX* (Farrugia, 2012 ▸).

**Table 2:** Selected inter­atomic distances (Å) and angles (°) for **1** and **2**

**1**			**2**
I1⋯S2	3.158 (7)	C10—S1	1.800 (4)
C1—S1	1.708 (5)	C1—N1	1.344 (7)
C6—I1	2.107 (4)	C5—S1	1.743 (5)
F2⋯H3	2.583 (7)		
			
C4—N1—C3	121.7 (4)	S1—C10—S1^i^	116.3 (4)
F1—C7—C6	120.1 (4)	N1—C1—C6	120.4 (5)
C7—C6—I1	121.0 (3)	C5—S1—C10	104.89 (19)

Symmetry code: (i) *x*, −*y* + 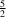 , −*z* + 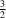 .
